# To bin or not to bin: why parasite abundance data should not be lumped into categories for statistical analysis

**DOI:** 10.1017/S003118202500040X

**Published:** 2025-03

**Authors:** Robert Poulin

**Affiliations:** Department of Zoology, University of Otago, Dunedin, New Zealand

**Keywords:** aggregation, continuous data, correlation, count data, inference, prevalence

## Abstract

The impact of macroparasites on their hosts is proportional to the number of parasites per host, or parasite abundance. Abundance values are count data, i.e. integers ranging from 0 to some maximum number, depending on the host–parasite system. When using parasite abundance as a predictor in statistical analysis, a common approach is to bin values, i.e. group hosts into infection categories based on abundance, and test for differences in some response variable (e.g. a host trait) among these categories. There are well-documented pitfalls associated with this approach. Here, I use a literature review to show that binning abundance values for analysis has been used in one-third of studies published in parasitological journals over the past 15 years, and half of the studies in ecological and behavioural journals, often without any justification. Binning abundance data into arbitrary categories has been much more common among studies using experimental infections than among those using naturally infected hosts. I then use simulated data to demonstrate that true and significant relationships between parasite abundance and host traits can be missed when abundance values are binned for analysis, and vice versa that when there is no underlying relationship between abundance and host traits, analysis of binned data can create a spurious one. This holds regardless of the prevalence of infection or the level of parasite aggregation in a host sample. These findings argue strongly for the practice of binning abundance data as a predictor variable to be abandoned in favour of more appropriate analytical approaches.

## Graphical abstract

**Figure:**
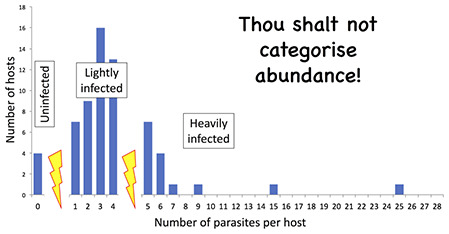

## Introduction

The impact of parasites on the physiology, growth, behaviour, reproductive success or survival of a host is generally proportional to the severity of infection. While quantifying the severity of infection for microparasites (*sensu* Lafferty and Kuris, 2002) relies on a range of indirect methods, for macroparasites (parasitic helminths and arthropods) the number of individual parasites of a particular species in/on an individual host, or parasite abundance, can usually be determined precisely and represents the most direct and appropriate measure of infection level. Parasite abundance ranges from 0 to some maximum number, depending on the host–parasite system under study. The magnitude of the parasites’ impact on host health usually increases with increasing abundance of infection (e.g. Anderson and Gordon, 1982; Benesh, 2011), though not necessarily in a linear fashion.

Typically, within a sample of hosts taken from a natural population, parasite abundance values show a highly skewed aggregated distribution with an excess of 0 or low values relative to high ones (Shaw and Dobson, 1995; Poulin, 2013). In other words, most host individuals harbour few or no parasites, whereas few host individuals harbour many parasites. This observed distribution of abundance values is best approximated by a negative binomial distribution, or in cases of high prevalence (high proportion of hosts harbouring at least 1 parasite) by a Poisson distribution. Count data with such distributions are not always amenable to traditional parametric statistical tests (e.g. ANOVA and regression) because they violate central assumptions of such tests. One popular solution in the past has been to use a logarithmic transformation to ‘normalize’ the distribution of abundance values prior to analysis, and thus decrease the influence of extreme outliers (a few high abundance values) (Fulford, 1994; Wilson et al., 1996). However, this approach is generally not recommended for a range of statistical reasons (Wilson et al., 1996; O’Hara and Kotze, 2010). Another approach has been to create classes or categories of infection levels by binning values. For example, in a sample where abundances range from 0 to 15 parasites per individual host, one could lump hosts into 3 categories: for example, those with 0 parasite, those with 1–5 parasites, and those with 6–15 parasites. The 3 categories (no infection, light infection, heavy infection) overcome the problems associated with the non-normal distribution of abundance values and serve to subdivide the hosts into 3 groups, whose properties (growth rates, behavioural scores, etc.) can then be compared using standard tests such as ANOVA.

This latter approach is flawed on multiple levels, however. First, the categorization is arbitrary and not biologically justifiable. Why using 5-versus-6 parasites per host as a cut-off point between categories? There is nothing *a priori* special about these abundances or any other abundance values to justify the placement of the boundary; whatever cut-off value is chosen, the risk of biased results is high. Second, like the arbitrary categorization of continuous data (Taylor and Yu, 2002; Royston et al., 2006; Naggara et al., 2011), the binning of count data is associated with several statistical pitfalls, as recognized in other scientific disciplines (Towers, 2014; Pollet et al., 2024). These include loss of information about individual differences, reduced statistical power, inability to detect non-linear effects, and increased probability of type I and II errors (i.e. rejecting the null hypothesis when it is actually true, and failure to reject the null hypothesis when it is actually false, respectively). Binning also makes it difficult to compare results from 1 study with results from other studies, since different studies bin abundance values in different ways. For all these reasons, binning count data may lead to incorrect conclusions and should be discouraged.

In spite of earlier warnings against this practice (see Poulin, 2019), binning abundance data to create artificial categories has remained a common approach among studies in ecological and veterinary parasitology, especially when abundance is treated as a predictor variable, not as a response. Here, I first (i) quantify the frequency at which abundance data are binned into arbitrary categories among recent studies in parasitological, ecological and behavioural journals; and then (ii) provide examples from simulated infection data to show how binning abundance data into arbitrary categories can result in low statistical power and/or incorrect interpretations when testing for associations with host traits. Finally, I discuss ways to avoid the need for categorization, except in certain circumstances, and instead use the raw count data in analyses. I hope the arguments presented here will encourage parasitologists to avoid unjustifiable binning of abundance data and its associated pitfalls in future studies.

## Methods

### Literature review

For the first dataset, to quantify the frequency with which parasitologists bin abundance data into categories, I searched all issues of the following 5 journals published between 2010 and 2024 inclusively: *International Journal for Parasitology, International Journal for Parasitology–Parasites and Wildlife, Journal of Helminthology, Journal of Parasitology* and *Parasitology*. These journals were chosen because *a priori* they were known to publish relevant kinds of studies. Although not capturing the full set of relevant articles published during that period, these 5 journals provide a large and representative sample of the practices in the field. Studies based on faecal egg counts or other measures that provide only indirect and/or unreliable estimates of true parasite abundance were excluded. All correlational or experimental studies that used abundance (whether or not uninfected hosts were included) of a focal parasite species as a predictor variable were included; studies treating parasite abundance as a response variable were not considered, after a preliminary survey indicated that categorising abundance data was extremely rare in such cases. The statistical test used (e.g. correlation coefficient, linear regression, generalized linear model (GLM) when abundance was treated as a count variable; ANOVA, Kruskal–Wallis test, etc. when abundance values were binned) was not taken into account. Actual parasite count data had to be available for analysis for a study to be included; for instance, studies in which hosts were challenged with different parasite doses or treated versus not treated with anthelmintics, resulting in different average abundances between groups, but were not dissected later to quantify parasite abundance in each individual, were excluded (there were several such studies). Some studies considered separately different parasite species, and therefore contributed more than 1 data entry to the final dataset (Supplementary Table S1).

For the second dataset, since many studies in ecology and behaviour create categories out of continuous data (see Beltran and Tarwater, 2024), I quantified the frequency at which researchers in these areas bin parasite abundance data into categories when testing the relationship between infection and host traits. The following 10 journals were searched in late December 2024 for articles published between 2010 and 2024 inclusively: *Animal Behaviour, Behavioral Ecology, Behavioral Ecology and Sociobiology, Ecology, Ethology, Evolutionary Ecology, Journal of Animal Ecology, Journal of Evolutionary Biology, Oecologia* and *Oikos*. The ‘topic’ search was conducted in the Web of Science Core Collection using the journal names and the following search string: (‘effect* of parasite*’) OR (‘influence* of parasite*’) OR (‘impact* of parasite*’) OR (‘role of parasite*’). The search returned 63 articles, whose title and abstract were individually checked for relevance. The final dataset included 14 articles, including both correlational and experimental studies (Supplementary Table S2). Here again, some studies considered separately different parasite species, and these studies therefore contributed more than 1 data entry to the final dataset.

For each data entry in each of the 2 datasets, I recorded the following: (i) the parasite species; (ii) the higher taxon to which the parasite belonged, i.e. trematodes, cestodes, nematodes, copepods, etc.; (ii) the higher host taxon, i.e. fish, mammals, crustaceans, etc.; (iv) the number of individual hosts examined; (v) whether the parasite infections were natural or achieved by experimental exposure; (vi) the minimum, maximum and range of abundance values; (vii) whether or not abundance data were binned, and if so in how many bins and their boundaries; (viii) whether a justification was provided for binning, and if so what that was; and (ix) the year of publication, authors, journal name, volume and pages of the source article.

### Simulated datasets

The simulated datasets are not meant to capture all possible parasite distributions and effect sizes. Instead, they consist of realistic but hypothetical cases used to illustrate the possibility that binning parasite abundance values can either fail to detect a true underlying relationship between parasite abundance and some host trait, or lead to the conclusion that there is an effect of abundance where in fact there is none. Therefore, only selected permutations of the data are used here in order to highlight the pitfalls of data binning.

Three distributions of parasites among 60 hosts, which is a typical sample size in field surveys, were manually generated, the first (distribution A) with low prevalence and high aggregation, the second (distribution B) with moderate prevalence and aggregation and the third (distribution C) with high prevalence and low aggregation (Supplementary Table S3). All 3 distributions have the same mean abundance of 4.0 parasites per host and similar ranges of values. Different binning strategies were used for the 3 distributions. Two bins were created for the first distribution (because of low prevalence: uninfected vs infected), 3 bins were created for the second distribution (uninfected, lightly infected, heavily infected) and 3 bins were created for the last one (lightly, moderately and heavily infected) (Supplementary Table S3).

For each analysis, parasite abundance was related to a hypothetical host trait, with a normal distribution and in all cases the same mean value and standard deviation (10.0 ± 3.2) (Supplementary Table S4, Supplementary Figure S1). Two scenarios were considered for each of the 3 parasite distributions (see Supplementary Tables S5–S7). First, abundance values were matched with host trait values to produce a weak and non-significant correlation (Spearman rank correlation, *r*_s_ < 0.2). Second, host trait values were reshuffled to produce a moderate and significant correlation with parasite abundance (*r*_s_ between 0.3 and 0.4).

Overall, I therefore considered 2 possible correlations with host trait values for each of 3 parasite distributions, for a total of 2 × 3 = 6 scenarios. In each case, I performed a 1-way ANOVA on host trait values, with the abundance bins as categorical variables. Here, it is assumed that the correlation between parasite abundance and host trait values captures the true relationship between these variables, since it takes individual data points into account and is not plagued by any arbitrary categorization. Therefore, the significance of the ANOVA result was contrasted with that of the Spearman correlation, to illustrate that binning abundance values can produce a different outcome, and lead to different conclusions, than treating them as individual data points. Spearman correlations were used as a reference baseline since the review of the literature (see above) revealed their wide usage in parasitological studies; however, their findings regarding the significance of the parasite abundance versus host trait relationships were consistent with those of GLMs (data not shown).

## Results

### Literature review

The first dataset, which focused on the parasitological literature between 2010 and 2024, included 181 cases in which abundance of a focal parasite taxon was used as a predictor of either host traits or infection parameters of another parasite taxon, obtained from 110 studies. The majority of cases involved trematodes (47), nematodes (44) or cestodes (33), with the rest involving other parasite taxa (e.g. acanthocephalans, monogeneans, copepods, mites, ticks). Fish (80 cases) and mammals (36) were by far the most common host taxa, the rest of cases involving mainly birds, amphibians, reptiles, insects and crustaceans. With respect to the nature of the studies, 135 cases relied on natural parasite infections, whereas 46 performed experimental infections.

**Figure 1.**
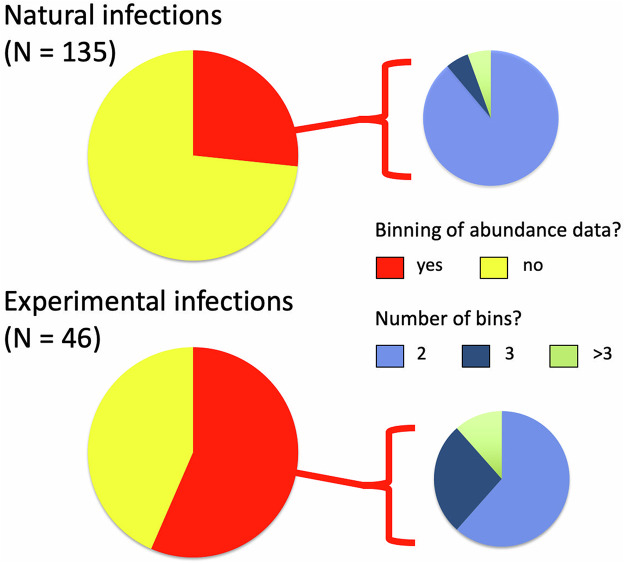
Proportions of studies in which parasite abundance was used as a predictor variable, where abundance values were used as individual counts (no binning) or instead lumped into categories of infection (binning). In the latter case, the proportion of cases where the data were split into 2 bins, 3 bins or more than 3 bins is also shown. The data come from studies published in parasitology journals between 2010 and 2024, and are shown separately for studies based on natural parasite infections and those that used experimental infections.

**Figure 2.**
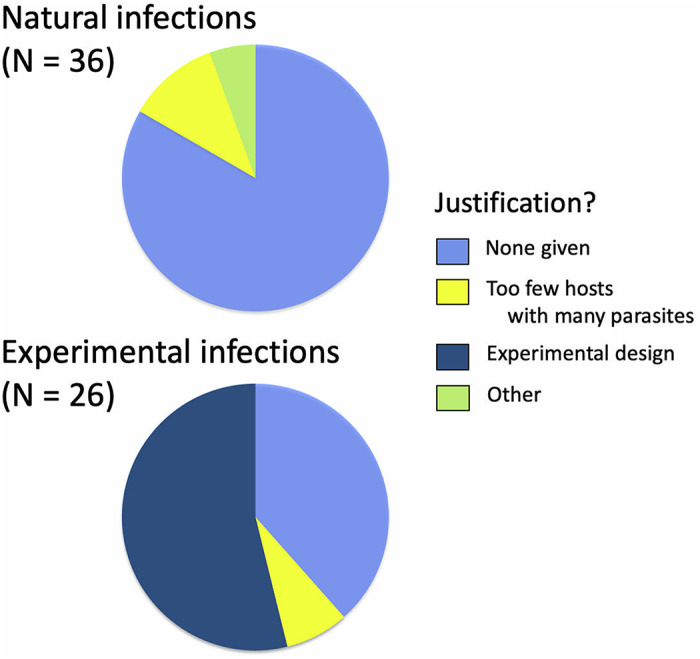
Reasons given by authors of studies in which parasite abundance was used as a predictor variable and where abundance values were lumped into categories of infection (binning). The data come from studies published in parasitology journals between 2010 and 2024, and are shown separately for studies based on natural parasite infections and those that used experimental infections.

Of the 181 cases, parasite abundance data were binned into categories in 62 cases, i.e. 34.3% of the time. The proportion of cases in which abundance data were binned was much higher among experimental studies than among those using natural parasite infections (Figure 1). For studies using natural infections, data were most frequently binned into 2 categories, usually infected versus non-infected, and rarely into 3 or more categories reflecting increasing numbers of parasites per host. In contrast, for studies using experimental infections, it was more common to divide hosts into 3 or more categories, reflecting the doses used for infection (e.g. low, moderate and high dose) (Figure 1). The authors of studies based on natural infections generally gave no reason or justification for lumping hosts with different parasite abundances into the same category; when they did, they usually explained that the low numbers of hosts infected by more than 1 or 2 parasites justified binning all infected hosts together (Figure 2). In contrast, authors of experimental studies generally appeared to use their experimental design, typically involving an unexposed control group and groups of hosts exposed to various doses of infective stages, as a reason for treating the hosts as distinct groups regardless of the actual numbers of parasites they acquired during the experimental exposure to infective stages (Figure 2).

**Figure 3.**
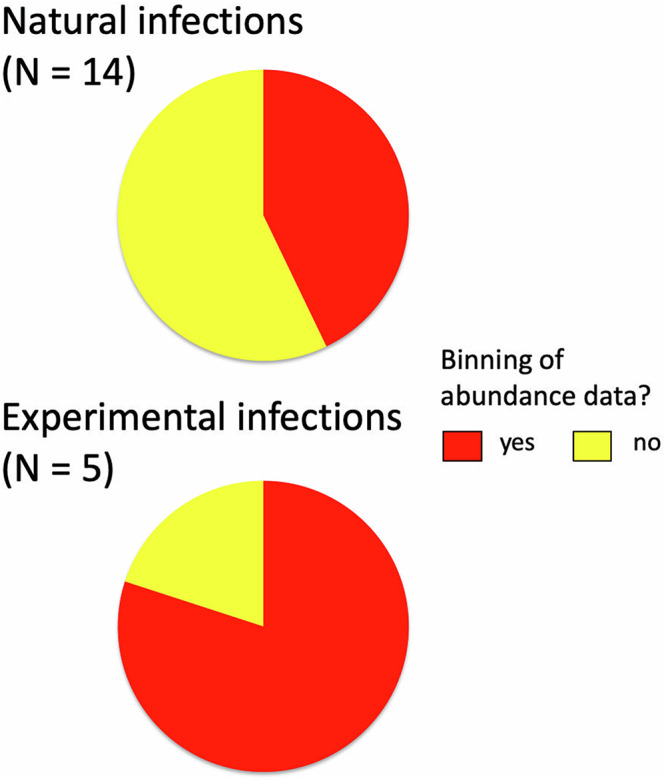
Proportions of studies in which parasite abundance was used as a predictor variable, where abundance values were used as individual counts (no binning) or instead lumped into categories of infection (binning). The data come from studies published in journals of ecology and animal behaviour between 2010 and 2024, and are shown separately for studies based on natural parasite infections and those that used experimental infections.

The second dataset, focusing on the ecological and behavioural literature from 2010 to 2024, included only 19 cases in which abundance of a focal parasite taxon was used as a predictor of host traits, obtained from 14 studies. These involved a range of parasite (helminths and arthropods) and host taxa, with none more frequently used than the others. Overall, parasite abundance data were binned into categories in 10 cases, i.e. 52.6% of the time. The proportion of cases in which abundance data were binned was much higher among the 5 experimental studies than among the 14 studies using natural parasite infections (Figure 3). Regardless of whether infections were natural or experimental, abundance data were binned into 2 categories, i.e. infected versus non-infected, in all but 1 case. In terms of justification, except in 1 case where no reason is given, the authors of these studies explained their decision to bin abundance values into categories on the basis of their experimental design or for statistical convenience (e.g. low prevalence – therefore, the few infected hosts had to be pooled into a single ‘infected’ category).

For both datasets, there was no obvious link between host sample size and the range of abundance values recorded in each case and whether or not the abundance data were binned and if so in how many bins. However, a weak temporal trend (i.e. a slight decrease in the annual frequency of data binning from 2010 to 2024) was apparent. Indeed, among studies published in parasitological journals, the proportion of cases where abundance data were binned was 38.6% (22 out of 57 cases) in the first 5 years (2010–2014), and only 25.5% (14 out of 55 cases) in the last 5 years (2020–2024).

### Simulated data

**Figure 4.**
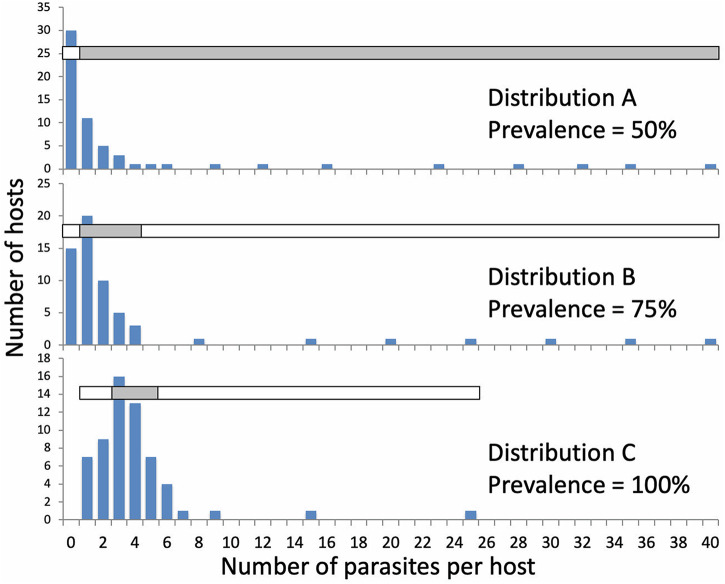
Three simulated distributions of parasite abundances among 60 host individuals. In each case, the total number of parasites in the sample is 240, with a mean abundance of 4.0 parasites per individual host. Note the different scales on the *y*-axes. The horizontal bars with alternating shading indicate how abundance values were lumped into 2 bins (infected and non-infected; distribution A) or 3 bins (distributions B and C).

I considered 3 hypothetical distributions of parasite abundance among 60 individual hosts (Figure 4), in scenarios where abundance is or is not significantly correlated with a host trait, to illustrate how binning abundance values may influence whether or not the correlation is detected. For distribution A, binning consisted of dividing the hosts into non-infected and infected. Several binning schemes, i.e. different ways of lumping hosts with different parasite abundances, were trialled for distributions B and C to produce 3 categories, lightly or not infected, moderately infected, and heavily infected, each containing enough host individuals for comparison by ANOVA. For each distribution, the chosen scheme (see Figure 4) is one of many which produced similar findings.

Analysis of the simulated datasets revealed that when treating parasite abundance as a categorical variable (by binning values), it is possible to obtain non-significant differences among groups based on ANOVA in situations in which abundance is actually significantly correlated with host trait values (Table 1). Similarly, it is possible to obtain significant effects of parasite abundance on host traits with an ANOVA on binned data in situations where these variables are not significantly correlated (Table 1). This is true regardless of parasite prevalence or the type of distribution of abundance values, from low to high aggregation among individual hosts (Table 1; see also Supplementary Tables S5–S7).

**Table 1.** Results of 2 alternative ways of analysing the association between parasite abundance and a hypothetical host trait, for 3 different distributions of parasites among a sample of 60 hosts (see Figure 4)

	Correlation with host trait		Treating parasite abundance as categories		
Parasite distribution	*r*_s_	*P*-value	Bins (range in no. parasites per host)	*F*-ratio	*P*-value
Distribution A (prevalence = 50%, high aggregation)	0.192	0.141	Uninfected (0), infected (1−40)	***F*_1,58_ = 4.63**	**0.035**
	**0.293**	**0.027**	Uninfected (0), infected (1−40)	*F*_1,58_ = 1.13	0.293
Distribution B (prevalence = 75%, medium aggregation)	0.168	0.199	Uninfected (0), lightly infected (1−4), heavily infected (8−40)	***F*_2,57_ = 8.08**	**0.0008**
	**0.358**	**0.005**	Uninfected (0), lightly infected (1−4), heavily infected (8−40)	*F*_2,57_ = 1.79	0.175
Distribution C (prevalence = 100%, low aggregation)	0.181	0.167	Lightly infected (1−2), moderately infected (3 − 5), heavily infected (6 − 25)	***F*_2,57_ = 10.25**	**0.0002**
	**0.391**	**0.002**	Lightly infected (1−2), moderately infected (3−5), heavily infected (6−25)	*F*_2,57_ = 1.357	0.266

The association was first established with a Spearman rank correlation coefficient (*r*_s_), then tested with a 1-way ANOVA (*F*-ratio) after abundance values were binned into either 2 categories (distribution A) or 3 categories (distributions B and C). Significant effects are shown in bold.

**Figure 5.**
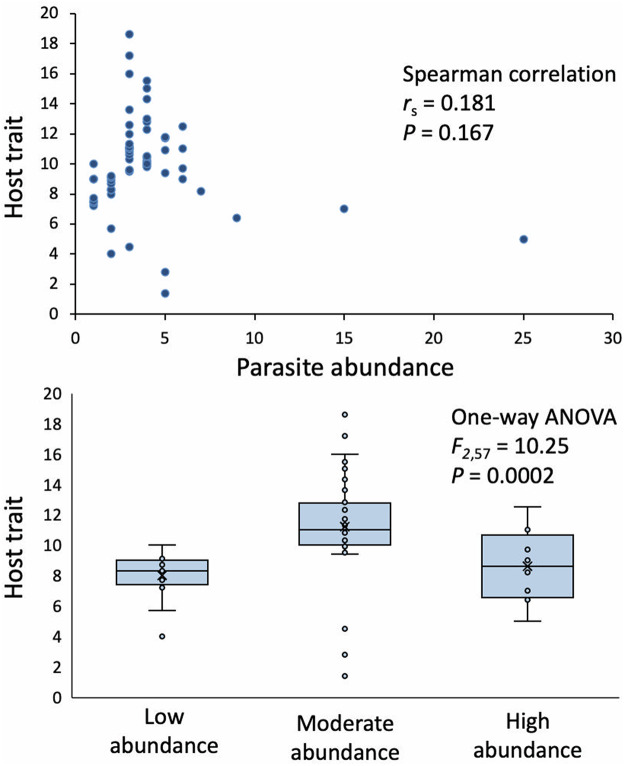
Association between simulated parasite abundance data (numbers of parasites per host; from distribution C in Figure 4) and a hypothetical host trait, measured both using a Spearman correlation coefficient (top) and a 1-way ANOVA after binning hosts into 3 groups based on their parasite abundance (bottom): low (1–2 parasites/host), moderate (3–5 parasites/host) and high (6–25 parasites/host). Whereas the correlation indicates a non-significant association, the ANOVA suggests there is one.

**Figure 6.**
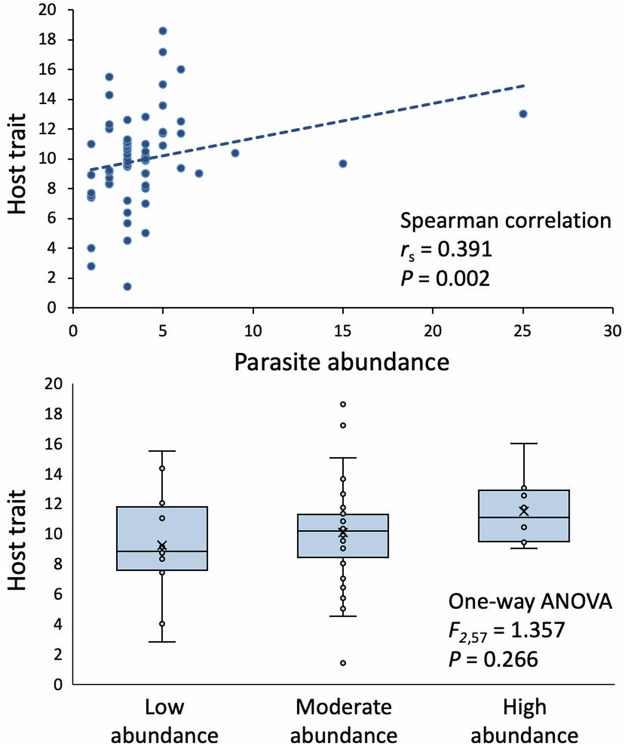
Association between simulated parasite abundance data (numbers of parasites per host; from distribution C in Figure 4) and a hypothetical host trait, measured both using a Spearman correlation coefficient (top) and a 1-way ANOVA after binning hosts into 3 groups based on their parasite abundance (bottom): low (1–2 parasites/host), moderate (3–5 parasites/host) and high (6–25 parasites/host). Whereas the correlation indicates a significant positive association, the ANOVA finds no association.

For example, using parasite abundance values from distribution C, it is possible to have a non-significant correlation between abundance and the host trait, and obtain significant differences based on an ANOVA when the same abundance values are binned into 3 groups and treated as a categorical variable (Table 1). In the example considered here, the ANOVA suggests that moderately infected hosts have greater trait values than those with light infections (Figure 5). Similarly, again using distribution C, it is possible to have a significant positive correlation between abundance and the host trait, but no significant differences in trait values among groups of hosts binned based on their parasite abundance (Figure 6), despite the fact the very same data are used for the 2 alternative analyses.

## Discussion

Because parasites are almost always aggregated among host individuals (Shaw and Dobson, 1995; Poulin, 2013), parasite abundance data typically show a highly skewed distribution, such as the negative binomial. This has caused problems for inferential analysis because traditional parametric tests assume near-normal data distribution, at least for the response variable. For this and other reasons, when abundance is used as a predictor variable, many studies bin abundance values, i.e. they group hosts into arbitrary categories each defined by a range of abundance values, and then test for differences in some host trait among categories. Here, I determined how frequently binning of parasite abundance values has been used in studies published over the past 15 years, and then demonstrate how this approach can lead to erroneous interpretations.

The literature review revealed that one-third of parasitological studies, and half of ecological and behavioural studies, have lumped hosts into categories based on parasite abundance when the latter is used as a predictor in data analysis. The apparent difference between disciplines may simply be due to the lower number of studies found in ecological and behavioural journals. Regardless, these results indicate that binning abundance data is a very common analytical approach despite the recognized pitfalls associated with categorising either count predictor variables (Towers, 2014; Pollet et al., 2024) or continuous predictor variables (Taylor and Yu, 2002; Royston et al., 2006; Naggara et al., 2011). Studies in which hosts were experimentally infected (exposure to different doses of infective stages) or treated for parasite removal (administration of anthelmintic vs placebo) were more likely to bin abundance data for analysis than those using naturally infected and untreated hosts. This was done even when hosts were individually examined after the experiment and actual parasite abundance was determined for each host. The authors of these studies generally used their experimental design as a justification for ignoring actual abundance data and instead grouping hosts into categories. From a biological perspective, the abundance values at the boundaries between these categories have no special significance, making the categories artificial and subjective. Yet justification of breakpoints used to establish categories of hosts is critical for transparency and reproducibility. In contrast, the authors of studies using naturally infected hosts generally gave no reason for binning them based on abundance. When they did give a reason, it was usually because there were too few infected hosts and it seemed appropriate to group them for a comparison with uninfected hosts.

During the literature survey, many studies were excluded. Notably, a large number of experimental studies consisted of host groups either infected or not by parasites, or exposed to different parasite doses, but not later dissected for parasite counts. In such studies, the infection status (infected or not) or exposure doses served to group individual hosts into categories for analysis. Since actual intensities of infection were not determined and thus unavailable as potential predictor variable, these studies were not included in the present dataset of past studies. Therefore, the actual number of studies in which intensity of infection is treated as a categorical variable is greatly underestimated. Having said this, such studies represent situations where binning abundance values is an acceptable practice since the actual values are unknown.

The analyses of simulated datasets demonstrate that regardless of the prevalence of infection or the level of parasite aggregation in a host sample, grouping hosts into categories by binning abundance data can lead to incorrect conclusions. It is possible that a real effect of parasite infections on host traits goes undetected when the analysis is made on host categories instead of across individual hosts. This suggests that binning abundance values results in reduced statistical power to detect real but modest effects of infection, possibly because the sample size for the ‘heavy infection’ category is probably smaller than for other categories in most realistic cases. One could argue that the weak effects (Spearman correlation coefficient, *r*_s_ < 0.4) likely to be missed when binning abundance values are too small to be biologically meaningful anyway; however, it is possible that these small effects are relevant, and failing to detect them because of an inappropriate analysis would lead to incorrect conclusions.

The opposite situation can also arise: an analysis based on subjective host categories can suggest there is an effect of infection, where in fact there is no effect. Comparable results were obtained in a recent study using simulated data in which, instead of a count predictor variable, a continuous predictor variable was analysed with and without being categorized (Beltran and Tarwater, 2024). Although these findings are based on simulated data, they should nonetheless sound alarm bells.

It is worth reiterating that the simulated datasets and relationships between parasite abundance and host traits used here do not capture the full range of possibilities. They were selected specifically to show that binning abundance values into arbitrary categories can either mask a true underlying relationship, or produce a spurious one. Nevertheless, it is important to note that in preliminary trials, many reshuffling of host trait values associated with particular parasite abundances produced the same erroneous results, suggesting that binning abundance values will often lead to the wrong conclusion. However, this may depend on effect size. In this study, with modest effect sizes linking parasite abundance and host trait values (correlation coefficients between 0.3 and 0.4), many reshufflings of the data all resulted in failure to detect the relationship once abundance data were binned into categories. This proved much less frequent when using larger effect sizes (correlation coefficients >0.7), suggesting that a strong effect of parasite abundance on host traits may be less likely to be missed when abundance is treated as a categorical variable. However, since modest effect sizes of parasite infections on host behavioural and physiological traits are commonly reported (Poulin, 1994; McElroy and de Buron, 2014; Sánchez et al., 2018), the risk that binning abundance data for analysis leads to gross misinterpretation is a real one.

In some circumstances, binning abundance values can be both necessary and acceptable. As explained above, in an experiment where hosts are exposed to different infection or anthelmintic treatments and parasites are not actually counted post-experiment for whatever reason, grouping hosts into infection categories based on treatment is the only approach possible. There may be other situations where binning abundance values into distinct categories is either inevitable or preferable. For example, if counting macroparasites is not possible for any reason, and only the absence or presence of infection can be detected, then contrasting uninfected hosts with infected hosts may be the only approach possible. Similarly, if individual parasites cannot be counted but visual estimates of the severity of infection (light vs heavy) are possible, based on scars or other external signs, then again categorising the hosts based on this criterion is acceptable. Alternatively, if parasites can be counted accurately but the distribution of abundance values turns out to be clearly bimodal, for whatever reason, then a split into 2 categories would seem justifiable. These are exceptional cases, however, and in the vast majority of cases abundance values should not be binned into arbitrary categories. This is important to avoid loss of information about individual differences among hosts, to improve statistical power and to lower the probability of type I and II errors.

Simple alternatives to binning abundance data exist when these data are used as predictor variable. Notably, GLMs do not assume any particular distribution for predictor variables, whether they are continuous or count variables (Madsen and Thyregod, 2010). This form of analysis, long recommended for parasitological data where abundance is treated as a response variable (Wilson and Grenfell, 1997; Alexander, 2012), is also the most appropriate when abundance is used as a predictor variable. GLMs are now easy to implement in R (R Core Team, 2024) or a range of other statistical packages. They represent the best approach to handle parasite count data, and their growing use in parasitology will hopefully see the practice of binning abundance values being abandoned.

## Supporting information

Poulin supplementary material Poulin supplementary material

## Supplementary material

The supplementary material for this article can be found at https://doi.org/10.1017/S003118202500040X.
